# A Comparative Transcriptome Analysis Identifying FGF23 Regulated Genes in the Kidney of a Mouse CKD Model

**DOI:** 10.1371/journal.pone.0044161

**Published:** 2012-09-06

**Authors:** Bing Dai, Valentin David, Aline Martin, Jinsong Huang, Hua Li, Yan Jiao, Weikuan Gu, L. Darryl Quarles

**Affiliations:** 1 University of Tennessee Health Science Center, Medicine-Nephrology, Memphis, Tennessee, United States of America; 2 University of Tennessee Health Science Center, Orthopaedic Surgery, Memphis, Tennessee, United States of America; Universidade de Sao Paulo, Brazil

## Abstract

Elevations of circulating Fibroblast growth factor 23 (FGF23) are associated with adverse cardiovascular outcomes and progression of renal failure in chronic kidney disease (CKD). Efforts to identify gene products whose transcription is directly regulated by FGF23 stimulation of fibroblast growth factor receptors (FGFR)/α-Klotho complexes in the kidney is confounded by both systemic alterations in calcium, phosphorus and vitamin D metabolism and intrinsic alterations caused by the underlying renal pathology in CKD. To identify FGF23 responsive genes in the kidney that might explain the association between FGF23 and adverse outcomes in CKD, we performed comparative genome wide analysis of gene expression profiles in the kidney of the Collagen 4 alpha 3 null mice (Col4a3^−/−^) model of progressive kidney disease with kidney expression profiles of Hypophosphatemic (Hyp) and FGF23 transgenic mouse models of elevated FGF23. The different complement of potentially confounding factors in these models allowed us to identify genes that are directly targeted by FGF23. This analysis found that α-Klotho, an anti-aging hormone and FGF23 co-receptor, was decreased by FGF23. We also identified additional FGF23-responsive transcripts and activation of networks associated with renal damage and chronic inflammation, including lipocalin 2 (Lcn2), transforming growth factor beta (TGF-β) and tumor necrosis factor-alpha (TNF-α) signaling pathways. Finally, we found that FGF23 suppresses angiotensin-converting enzyme 2 (ACE2) expression in the kidney, thereby providing a pathway for FGF23 regulation of the renin-angiotensin system. These gene products provide a possible mechanistic links between elevated FGF23 and pathways responsible for renal failure progression and cardiovascular diseases.

## Introduction

FGF23 is a bone-derived hormone that regulates phosphate and vitamin D metabolism through FGFR/α-Klotho co-receptors [1] that are expressed in a limited number of tissues, including the kidney [2]. In the kidney, FGF23 suppresses sodium-phosphate co-transporter function leading to phosphaturia and reduces 1,25(OH)_2_D synthesis in the proximal tubule [3], [4]. Physiologically, FGF23 is part of a bone-kidney feedback loop [4], [5], where circulating 1,25(OH)_2_D stimulates FGF23 production in bone and FGF23 suppresses 1,25(OH)_2_D production in the kidney [5]. FGF23 expression is also regulated by local bone-derived factors that may link bone mineralization with renal phosphate handling [6], [7], [8].

FGF23 plays a pathological role in hereditary hypophosphatemic disorders [8] and tumor induced osteomalacia [9]. Elevations of circulating FGF23 also occur early in the course of chronic kidney disease (CKD), where it stimulates phosphaturia to maintain phosphate balance and contributes to the development of secondary hyperparathyroidism through suppression of 1,25(OH)_2_D levels [10], [11], [12]. FGF23 is also markedly elevated in patients with end stage renal disease (ESRD) [13], [14], [15].

Elevated FGF23 levels are associated with left-ventricular hypertrophy and hypertension in patients with X-linked hypophosphatemia (XLH) [16]. FGF23 is also an independent risk factor for left ventricular hypertrophy [17] and cardiovascular disease [18] in the general population. In chronic kidney disease, FGF23 is one of the strongest predictors of mortality [19], [20], and adverse cardiovascular outcomes [21], [22]. In addition, elevated circulating FGF23 concentrations are independently associated with more rapid progression of kidney disease [23] and renal allograft loss [24].

There are many gaps in our knowledge of the molecular mechanisms whereby FGF23 regulates kidney function and leads to adverse outcomes in CKD. It is uncertain which tubular segment and FGF receptors mediate the effects of FGF23 on the kidney [25]. In addition, knowledge of the full complement of renal gene products regulated by FGF23 in the kidney that might mediate progressive renal damage or kidney processes affecting cardiovascular disease is largely unexplored. Without this information, it remains uncertain whether the associations between FGF23 and adverse outcomes represent cause-and-effect relationships or epiphenomena due to co-variance of FGF23 with other causative factors arising from the loss of renal function [26], [27]. In addition, because of the limited number of organs that co-express FGFR/−α-Klotho complexes [28], it is also possible that elevated circulating FGF23 are directly mediated by off-target effects of FGF23 to activate FGF receptors in non-renal tissues [22], rather than indirectly thru FGFR/α-Klotho-dependent modulation of systemic pathways affecting the cardiovascular system.

Determining the FGF23 responsive genes in the kidney in the setting of chronic kidney disease is challenging because of the systemic effects resulting from FGF23 regulation of phosphate and vitamin D homeostasis and the intrinsic abnormalities related to kidney disease process. To define FGF23 responsive genes in CKD, we performed a genome wide comparative analysis of kidney gene expression in the Col4a3^−/−^ model of excess FGF23 [29] and CKD. We compared this CKD model to the kidney gene transcriptome of models of excess FGF23 without CKD that have different abnormalities of phosphate and vitamin D regulation [30], [31]. Shared candidate FGF23 responsive genes in the kidney of these models were confirmed by assessing their expression in FGF23^−/−^ mice and following the acute and chronic administration of recombinant FGF23 *in vivo.* Direct regulation of a subset of genes by FGF23 was assessed in distal tubule cells *ex vivo*. We identified several genes regulated by FGF23 that may link this hormone to processes responsible for progression of kidney disease as well as pathways responsible for adverse cardiovascular outcomes.

## Results

### Col4a3^−/−^ Mice, a Model of FGF23 Excess

**Figure 1:**
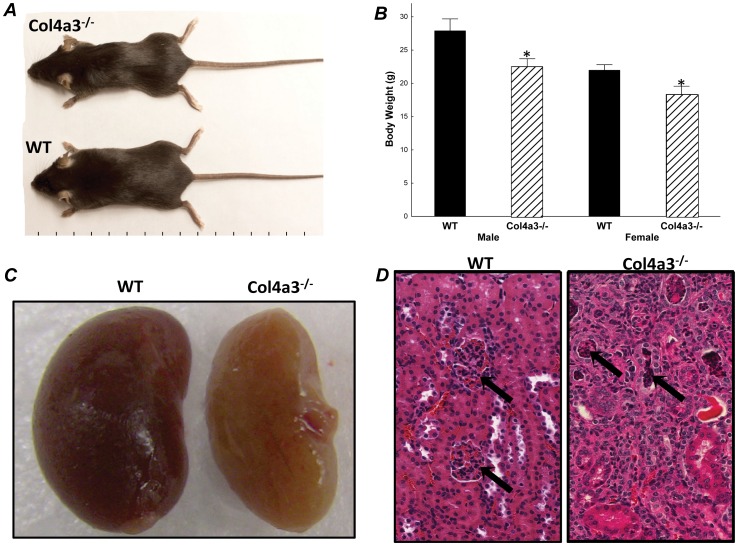
(A) Gross appearance and (B) body weight of 12 week-old wild-type (WT), and Col4a3−/− mice. (**C**) Kidney morphology showing reduced perfusion and (**D**) H&E renal histology showing glomerulosclerosis in the Col4a3^−/−^ animals. Values are expressed as mean±SEM, P<0.05 vs: (*) WT, n≥13 mice/group.

Col4a3^+/+^(WT) Col4a3^+/−^ and Col4a3^−/−^ mice were found to be born with the expected Medelian frequency. Homozygous Col4a3^−/−^ display are known to display a progressive decrease in kidney function [32]. By 12 weeks-of-age, we observed a decrease in body-weight in Col4a3^−/−^ mice (**Figure 1A &B**) and the presence of kidney disease, as evidenced by reduced kidney size (**Figure 1C**) and histological evidence of glomerulosclerosis and interstitial cell infiltration (**Figure 1D**). A 3-fold increase in blood urea nitrogen (BUN) and 2-fold increase in creatinine were observed in Col4a3^−/−^ mice (**Table 1**). Col4a3^−/−^ mice had a 55-fold increase in serum PTH and a 9-fold increase in serum FGF23 concentrations along with an increase in fractional excretion of phosphate. Serum phosphate and calcium concentrations were also increased in Col4a3^−/−^ mice (**Table 1**).

**Table 1:** Serum biochemistry of WT and Col4a3−/− mice.

	WT	Col4a3^−/−^
BUN (mg/dL)	20.29±0.67	59.01±8.59*
Creatinine (mg/dL)	0.45±0.02	0.82±0.15*
FGF23 (pg/mL)	137.34±9.54	1248.29±188.50*
PTH (pg/mL)	32.28±3.81	1772.18±452.94*
FEPi (%)	4.66±1.62	14.82±2.65*
PO4^−^ (mg/dL)	6.58±0.43	9.39±0.50*
Ca^2+^ (mg/dL)	8.88±0.33	9.38±0.29
ALP (IU/L)	67.16±7.52	87.79±10.56

Values are expressed as mean ± SEM from at least 13 mice per group. Comparisons were performed using one-way ANOVA and post-hoc Fisher test.BUN: Blood Urea Nitrogen; FEPi: Fractional Excretion of Phosphorus; PO4^−^: phosphorus; Ca ^2+^: total calcium; ALP: Alkaline Phosphatase. (*) P<0.05 vs. WT.

### Identification of Additional Renal Signalization Pathways in Chronic Kidney Disease

**Figure 2:**
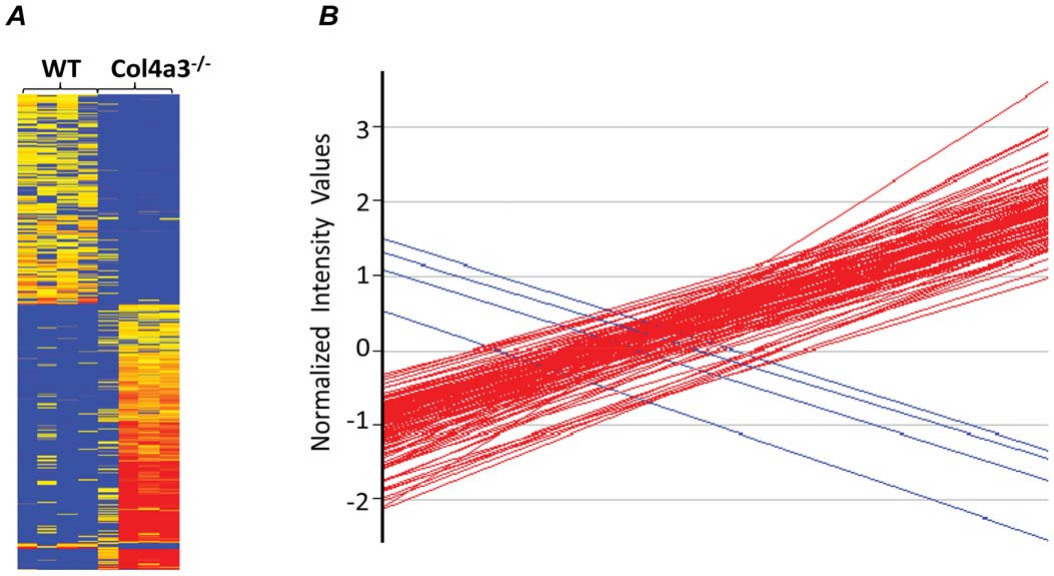
(A) Cluster analysis of microarray performed on Kidneys from 12 week-old wild-type (WT), and Col4a3^−/−^ mice. Gene expression is represented on the heat map from the less expressed (blue) to the more expressed (red). (**B**) Graphic representation of transcripts expressed at least five fold in Col4a3^−/−^ as compared to WT.

Clustering of all the significant genes revealed two different patterns corresponding to increased and decreased expression of renal transcripts in Col4a3^−/−^ mice as compared to their WT age-matched control animals (**Figure 2A**). Subsequent analysis revealed that chronic kidney disease led to a dramatic upregulation of gene transcripts, whereas the degree of downregulation was more limited. For instance, using a stringent, five-fold selection criteria to identify changes in gene transcripts, we found that only 4 transcripts were downregulated by this magnitude, whereas 500 genes were upregulated by at least 5-fold in Col4a3^−/−^ mice (**Figure 2B**). This shows that kidney disease progression involves activation of gene transcription and that modifications in the renal transcriptiome is not simply a passive process caused by loss of functioning renal tissue.

The top 25 upregulated genes (**Table 2**) showed evidence of matrix protein replacement with increased collagen synthesis (Col1a1 and Col3a1) and cellular infiltration (Cxcl1, Lyzs, Ccl5, Lyz2, Lyz, C3 VCAM1 and Ear2), consistent with the histological presence of chronic kidney disease in the mice. In total, more than 30 transcripts of protein belonging to the collagen familly were increased in the kidneys of Col4a3^−/−^ mice, as well as proteins from the TNFα superfamilly (30 transcripts) and TGFβ superfamilly (11 transcripts). Furthermore, TIMP1 was increased along with a substantial disregulation in proteases in the kidney of Col4a3^−/−^ mice, with a total of 24 mettaloendopeptidases being overexpressed.

**Table 2:** Expression fold change of the top 25 kidney genes up-regulated in Col4a3^−/−^ mice.

Gene Name	Symbol	Fold Change
lipocalin 2.	Lcn2	53.8
tissue inhibitor of metalloproteinase 1	Timp1	23.7
serine (or cysteine) peptidase inhibitor, clade A, member 3N.	Serpina3n	20.3
amiloride binding protein 1	Abp1	16.7
chemokine (C-X-C motif) ligand 1.	Cxcl1	14.4
lysozyme (Lyzs)	Lyzs	14.4
chemokine (C-C motif) ligand 5.	Ccl5	14.0
collagen, type I, alpha 1.	Col1a1	13.5
eosinophil-associated, ribonuclease A family, member 2.	Ear2	12.4
collagen, type III, alpha 1.	Col3a1	12.4
lysozyme 2	Lyz2	12.4
lysozyme.	Lyz	12.3
complement	C3	12.3
vascular cell adhesion molecule 1.	Vcam1	11.5
serine (or cysteine) peptidase inhibitor, clade A, member 10	Serpina10	9.3
chemokine (C-C motif) ligand 9	Ccl9	9.3
immunoglobulin lambda variable 1	Igl-V1	9.0
B-cell leukemia/lymphoma 2 related protein A1d	Bcl2a1d	8.4
CD44 antigen	Cd44	7.9
eosinophil-associated, ribonuclease A family, member 3	Ear3	7.8
matrix metallopeptidase 2.	Mmp2	7.7
CD14 antigen.	Cd14	7.6
ubiquitin D	Ubd	7.5
histone cluster 1, H2an	Hist1h2an	7.5
serine (or cysteine) peptidase inhibitor, clade A, member 3G	Serpina3g	7.3

Values were obtained after clustering analysis on microarray performed in kidney of *WT and Col4a3^−/−^ mice* (cluster is represented in Figure 2). n = 4 samples/group. Values are expressed as fold change compared to the WT control value. Genes were selected based on a P value threshold of 0.05 and a minimum fold-change absolute value of 2.

The top 25 down-regulated genes are shown in **Table 3**. Of note, we found evidence for reductions in DNAse1 and epidermal growth factor (EGF). In addition, we observed reductions in COP9 [33], which regulate ubiquitin-meidated proteoloysis of cullin that is cause of pseudohypoaldosteroinism type 2 and involved in distal tubular regulation of blood pressure and potasium homeostasis [34]. We also observed reduction in Cyp2c44, which important in producing compensatory renal artery vasodilation in response to salt-loading through the regulation of prostaglandin metabolism [35]. We also observed reduction in Slc6a19, which is a major luminal sodium-dependent neutral amino acid transporter in the proximal tubule [36] and parvalbumin, which is involved in distal convoluted sodium transport [37]. Higd1c, which belongs to hypoxia inducible genes that may play a role in protecting the kidney from hypoxic injury during progressive CKD [38], was also reduced in Col4a3^−/−^ kidneys. Corin, a protease that activates atrial natriuretic peptide, was also reduced in the kidneys of Col4a3^−/−^ mice [39].

**Figure 3:**
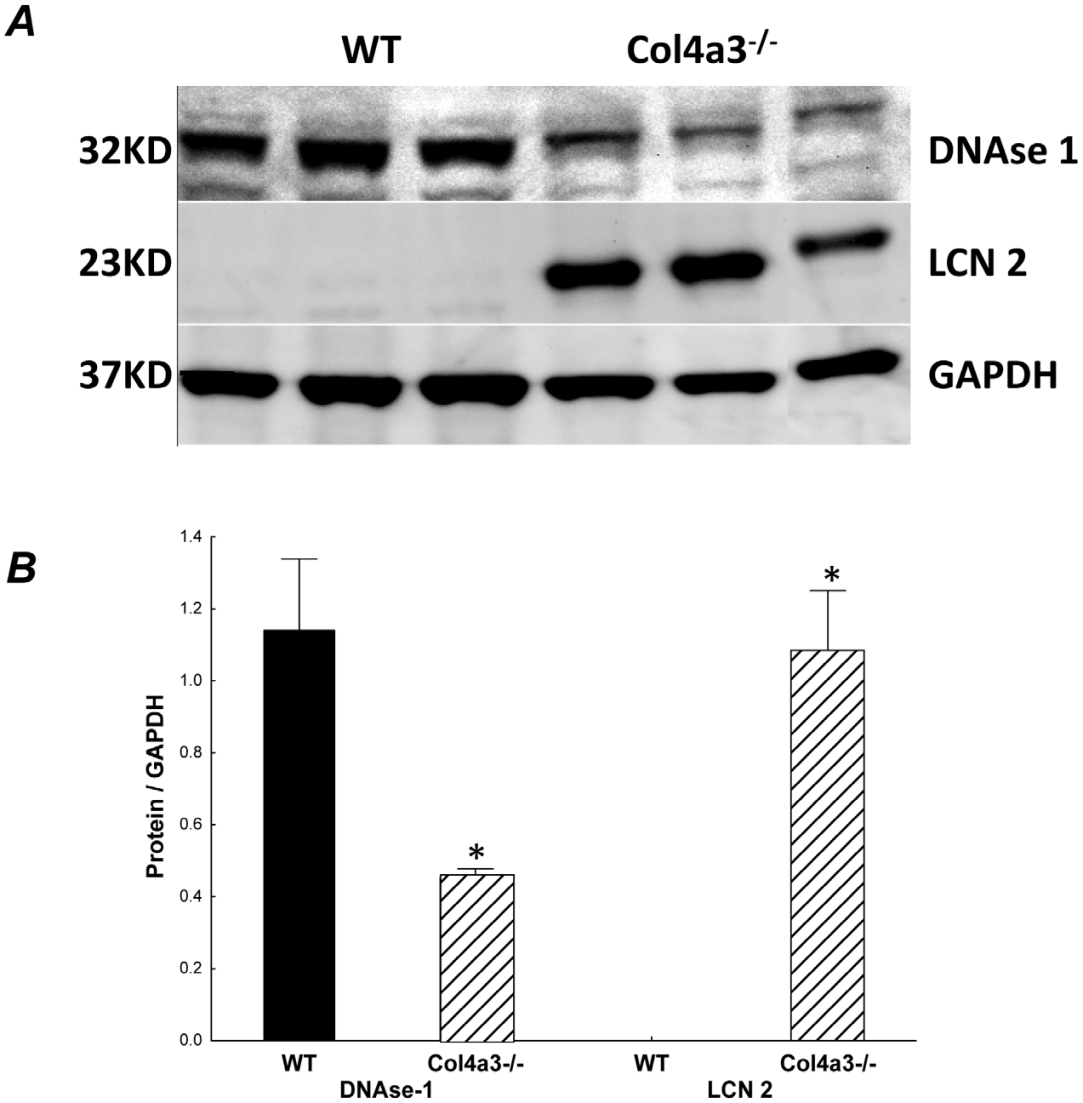
(A) Western blots and corresponding (B) quantification of the most upregulated and downregulated gene product in WT and Col4a3^−/−^ mice.

A total of twelve up-regulated and twelve downregulated genes were randomly chosen from the renal Co4a3^−/−^ transcriptome to be confirmed by RT-PCR as shown in **Table 4**. We also confirmed that the proteins encoded by the mRNAs of the most downregulated and upregulated genes, DNAse1 and Lcn2 respectively, were also altered, as shown in **Figure 3**.

**Table 3:** Fold-change of the top 25 down-regulated genes in Col4a3^−/−^ mice.

Gene Name	Symbol	Fold Change
deoxyribonuclease I.	Dnase1	**−**8.4
minichromosome maintenance deficient 6	Mcm6	**−**7.2
hemoglobin, beta adult minor chain	Hbb-b2	**−**7.2
cytochrome P450, family 2, subfamily d, polypeptide 12	Cyp2d12	**−**6.8
COP9 (constitutive photomorphogenic) homolog, subunit 8.	COP9	**−**4.9
4-hydroxyphenylpyruvic acid dioxygenase.	Hpd	**−**4.8
hemoglobin, beta adult major chain	Hbb-b1	**−**4.6
cytochrome P450, family 2, subfamily c, polypeptide 44	Cyp2c44	**−**3.6
erythroid delta-aminolevulinate synthase 2	Alas2	**−**3.6
solute carrier family 6 (neurotransmitter transporter	Slc6a19	**−**3.5
epidermal growth factor	Egf	**−**3.3
parvalbumin	Pvalb	**−**3.2
camello-like 1	Cml1	**−**3.2
UDP glucuronosyltransferase 1 family, polypeptide A7C	Ugt1a7c	**−**3.0
G protein-coupled receptor 112	Gpr112	**−**3.0
ureidopropionase, beta	Upb1	**−**2.9
hypoxia inducible domain family, member 1C	Higd1c	**−**2.9
hydroxyacid oxidase (glycolate oxidase) 3.	Hao3	**−**2.8
bisphosphate 3'-nucleotidase 1	Bpnt1	**−**2.8
ureidopropionase, beta	Upb1	**−**2.8
sorbitol dehydrogenase	Sord	**−**2.7
endothelial cell-specific molecule 1	Esm1	**−**2.7
glycine N-methyltransferase	Gnmt	**−**2.7
adenylate kinase 3 alpha-like 1	Ak3l1	**−**2.7
corin	Corin	**−**2.7

Values were obtained after clustering analysis on microarray performed in kidney of *WT and Col4a3^−/−^ mice* (cluster is represented in Figure 2). n = 4 samples/group. Values are expressed as fold change compared to the WT control value. Genes were selected based on a P value threshold of 0.05 and a minimum fold-change absolute value of 2.

**Table 4:** Expression fold change of selected genes confirmed by RT-PCR in Col4a3^−/−^ and WT mice.

Upregulated genes			Down regulated genes		
GeneSymbol	Microarray	RT-PCR	GeneSymbol	Microarray	RT-PCR
FGF23 regulated genes involvedin mineral metabolism					
Cyp24a1	2.6	5.1	Npt2c	**−**2.0	**−**2.3
Cyp27b1	1.3 (NS)	1.1 (NS)	Npt2a	**−**1.4(NS)	**−**1.6(NS)
Genes significantly modifiedin microarray dataset					
Lcn2	53.8	313.3	Dnase1	**−**8.4	**−**7.8
Timp1	23.7	157.8	Hbb-b2	**−**7.2	**−**7.2
Vcam1	11.5	25.8	Cyp2d12	**−**6.8	**−**6.8
MGP	6.1	9.1	Hbb-b1	**−**4.6	**−**6.9
Adamts2	4.9	26.7	Cyp2c44	**−**3.6	**−**4.0
STAT3s1	2.4	4.6	Aqp 11	**−**2.4	**−**5.1
Slc34a2	4.0	7.9	Cyp 51	**−**2.3	**−**2.2
CFI	3.2	5.8	Car14	**−**2,5	**−**3.8
Pla2g7	1.7	6.5	Afm	**−**2.2	**−**1.9
Lgals3bp	2,1	18.7	Slca2	**−**1.6	**−**1.7

Values are expressed as fold change compared to the WT value. n = 4 samples/group. Comparisons were performed using Student T test. P<0.05 vs: WT.

### FGF23-related Gene Transcripts in the Kidney

**Figure 4:**
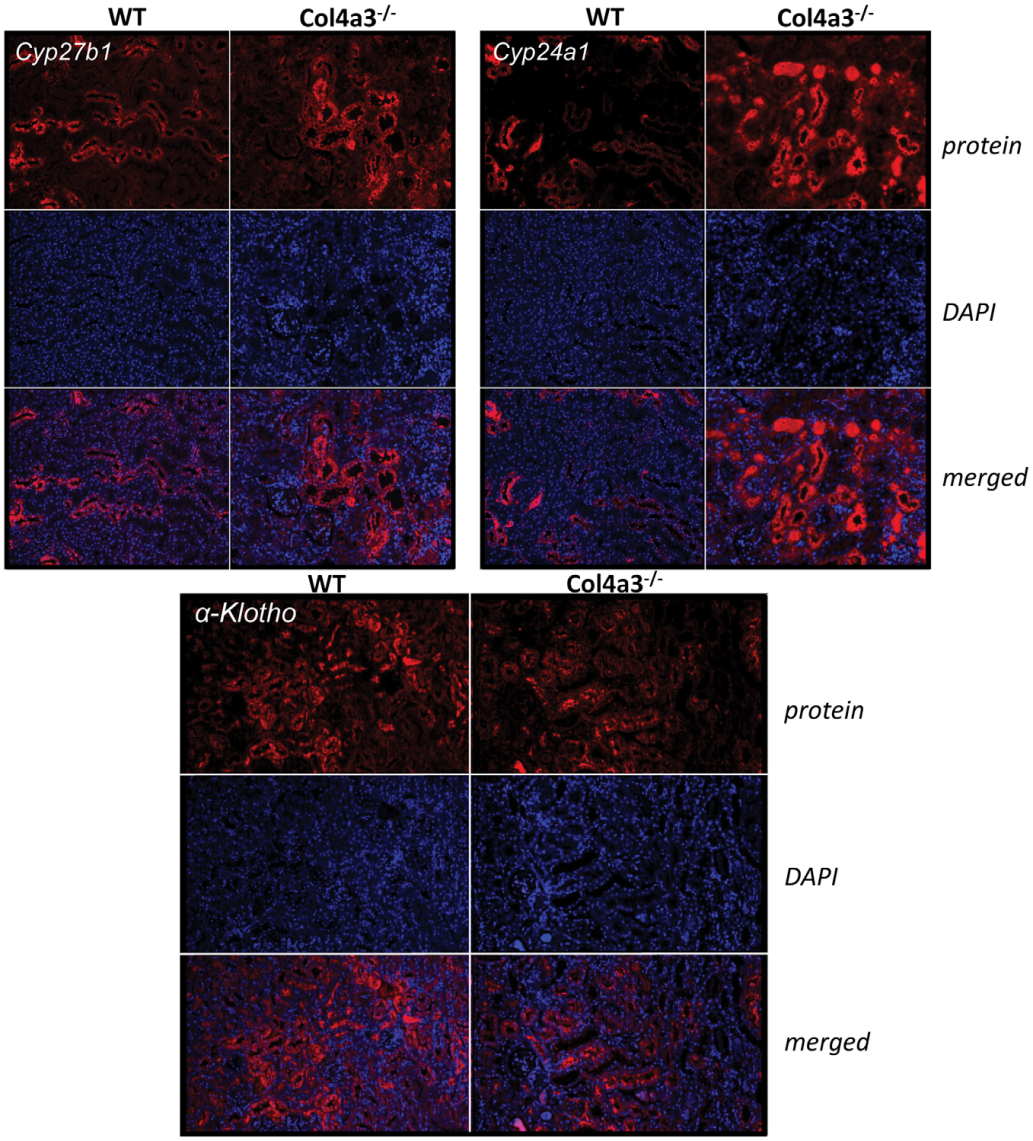
Immunohistochemistry of Cyp27b1, Cyp24a1 and α-klotho in the kidneys of WT and Col4a3^−/−^ mice.

To establish that the Col4a3^−/−^ microarray data set contained genes involved in FGF23 regulation of mineral metabolism, we initially focused on alterations in Cyp24a1, Cyp27b1, Npt2a, Npt2c and Klotho expression. We found that Col4a3^−/−^ mice displayed an increase in the renal Cyp24a1 transcripts, (2.6 and 5.1 fold by microarray and RT-PCR, respectively) as well as marked increase in Cyp24a1 protein level (**Figure 4**). However, we failed to detect any significant changes in Cyp27b1 expression. Additionally, Npt2c (**−**2.0 and **−**2.3 fold by microarray and RT-PCR), but not Npt2a, was down-regulated in the kidney of Col4a3^−/−^. Most importantly, α-Klotho, the FGF23 co-receptor, was down-regulated (**−**2 and **−**2.2 fold by microarray and RT-PCR) and α-Klotho protein levels in the kidney were reduced by immunohistochemical staining (**Figure 4**).

### Comparative Analysis of FGF23 Excess Models

To determine additional FGF23-responsive genes in the kidney of Col4a3^−/−^ mice, we compared microarray analysis of kidneys isolated from 12 week-old WT and Col4a3^−/−^ mice with the renal transcriptome in Hyp mice, which have hypophosphatemia and elevated FGF23 caused by inactivating mutations of Phex in osteoblasts [40], and FGF23 transgenic mice [31]. We hypothesized that shared genes in these three data sets would be enriched with FGF23-responsive transcripts.

**Figure 5:**
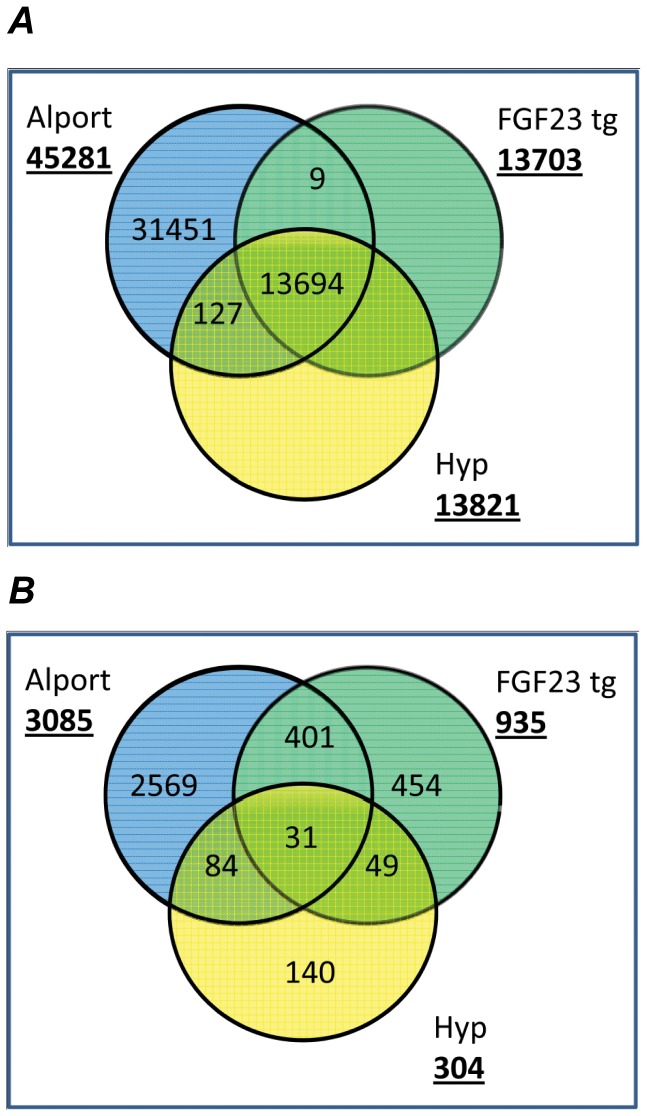
Venn diagram of (A) the total number of genes detected in all 3 data sets and (B) significantly regulated genes in all 3 models.

From 13694 transcripts present in all three datasets, 31 were found to be significantly altered in the kidney of two or more mutant mice models compared to their respective WT control mice (**Figure 5**). We have identified 19 of these genes that were consistently (downregulated or upregulated compared to their respective controls in all three datasets) altered in Col4a3^−/−^, Hyp and FGF23-transgenic mice (**Table 5**) by subsequently testing by PCR other populations of the same mice. Eleven gene transcripts were increased (**Table 5**), including lipocalin 2 (Lcn2), which was the most up-regulated transcript common to Col4a3^−/−^ and FGF23tg databases (but not the Hyp data set). In addition, inflamatory markers, including VCAM1, which is expressed in proximal tubule cells in response to inflammatory renal diseases [41], complement factor I, a serine protease that regulates the complement cascacade, and galectin-3-binding protein (LGALS3BP), were increased in all data sets. Several genes related to cell signaling were also increased, including tumor-associated calcium signal transducer 2 (Tacstd2), Receptor activity modifying protein 2 (Ramp2), guanylate binding protein 2, immediate early response 3, (ler3), phospholipase A2 (Pla2g7), phospholipid scramblase 1 (Plscr1). Lipoprotein-associated phospholipase A2 (Pla2g7), an enzyme mostly synthesized by plaque inflammatory cells (macrophages, T cells, mast cells) that hydrolyzes oxidized phospholipids in LDL was also upregulated.

**Table 5:** Expression fold change of 19 renal genes modified in models of excess FGF23.

Gene Name	Symbol	Col4a3−/−	Hyp	FGF23tg
**Increased**				
lipocalin 2	Lcn2	53.8	NM	2.0
vascular cell adhesion molecule 1	Vcam1	11.5	1.4	1.4
complement component factor i	Cfi	3.2	3.2	1.6
tumor-associated calcium signal transducer 2	Tacstd2	2.5	1.3	1.4
lectin, galactoside-binding, soluble, 3 binding protein	Lgals3bp	2.1	1.3	1.3
phospholipase A2, group VII	Pla2g7	1.7	1.9	2.0
immediate early response 3	Ier3	1.6	1.8	2.2
transporter 1, ATP-binding cassette, sub-family B	Tap1	1.6	1.5	1.2
receptor (calcitonin) activity modifying protein 2	Ramp2	1.5	1.5	1.6
phospholipid scramblase 1	Plscr1	1.4	1.4	1.3
guanylate binding protein 2	Gbp2	1.3	1.6	1.3
**Decreased**				
deoxyribonuclease I	Dnase1	**−**8.4	**−**4.3	**−**5.0
carbonic anhydrase 14	Car14	**−**2.5	**−**1.6	**−**1.7
Afamin	Afm	**−**2.2	**−**1.6	**−**1.5
Klotho	Kl	**−**2.0	**−**1.4	**−**1.6
abhydrolase domain containing 14A	Abhd14a	**−**1.9	**−**1.5	**−**1.3
angiotensin I converting enzyme 2	Ace2	**−**1.8	**−**2.3	**−**1.9
solute carrier family 2, member 2	Slc2a2	**−**1.6	**−**1.4	**−**1.3
thioesterase superfamily member 2	Them2	**−**1.6	**−**1.4	**−**1.2

Values are expressed as mean±SEM and as a relative percentage of the respective WT control value. Comparisons were performed using Student T test. P<0.05 vs: WT. NM, gene not present in the dataset.

With regards to down-regulated genes, 8 were reduced in all three data sets. Most interestingly, in addtion to reductions in α-Klotho described above, we also found that DNase1, a secreted nuclease that eliminates DNA from necrotic cells, was dramatically reduced in all three data sets. Most interestingly, angiotensin-converting enzyme (ACE) 2, a homolog to the carboxypeptidase ACE, was decreased in all three data sets. Finally, Them2 (thioesterase superfamily member 2) a 140-amino-acid protein of unknown biological function was also decreased.

**Figure 6:**
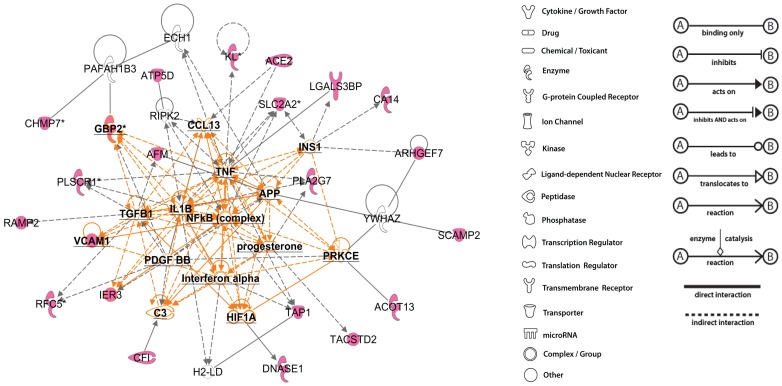
Ingenuity Pathway analysis (IPA) performed on 31 listed genes. The network is built according the identified interconnected pathways involving the highest majority of genes. Genes represented in pink color belong to the cluster. Genes represented in bold font are central regulators of the identified pathways that do not belong to the cluster. Genes represented in white color are other intermediary regulators that do not belong to the cluster.

Finally, we performed an Ingenuity Pathway Analysis to identify molecular interactions networks (**Figure 6**) related to these newly identified transcripts. Consistent with the non-mineral metabolism pattern of the expanded set of FGF23-regulated genes, this analysis suggests a central role of activation of transforming growth factor beta and tumor necrosis factor alpha (TGF-beta and TNF- alpha), nuclear factor of kappa light polypeptide gene enhancer in B-cells 1 (NFkB), interleukin 1, beta (IL1B), interferon, platlet derived growth factor (PDGF), progesterone, protein kinase C, epsilon (PRKCE), and Chemokine (C-C motif) ligand 13 (CCL13) pathways in the common genes regulated in the three data sets, consistent with activation of inflamatory and immunoregulatory processes.

### Independent Confirmation of Newly Identified FGF23-responsive Genes

We have used complementary *in vivo* approaches to verify FGF23 regulated genes in the kidney. First, we tested the effects of chronic daily administration of rat recombinant rFGF23 on genes identified from the comparative microarray analysis, plus additional genes (Cyp24a1, Cyp27b1, Npt2a and Npt2c) in FGF23^−/−^ and compound Col4a3^−/−^ FGF23^−/−^ mice. The FGF23 null background was used to minimize the effects of endogenous FGF23 production peaks, as well as the amount of FGF23 injected to these animals. The administration of 50ng/g of rFGF23 twice daily to FGF23^−/−^ and compound Col4a3^−/−^ FGF23^−/−^ mice resulted in a ∼12-fold increase in Cyp24a1 and induced a decrease in Cyp27b1 expression (**Table 6**), consistent with known actions of FGF23 on these gene products. Additionally, we have found that chronic FGF23 administration induced elevations in 5 genes (lcn2, cfi, vcam1,gbp2 and plscr1) and decreased the expression of 4 genes (dnase1, car14 ace2 and slca2) in FGF23^−/−^ mice.

**Table 6:** Expression fold change of renal genes modified after injection during 8 weeks of rFGF23.

Gene Name	Symbol	Fgf23^−/−^	Col4a3^−/−^ Fgf23^−/−^	Col4a3^−/−^ Fgf23^−/−^
		+ rFGF23		+ rFGF23
**FGF23 regulated genes involved in mineral metabolism**				
cytochrome P450, family 24, subfamily A, polypeptide 1	Cyp24a1	+12.6	+1.5	+12.5
cytochrome P450, family 27, subfamily B, polypeptide 1	Cyp27b1	**−**7.7	+1.3	**−**3.9
solute carrier family 34 (sodium phosphate), member 3	Npt2c	**−**1.2 (NS)	**−**1.8	**−**2.6
solute carrier family 34 (sodium phosphate), member 1	Npt2a	**−**1.2 (NS)	1.0 (NS)	**−**1.2 (NS)
**Increased in microarray comparative analysis**				
Lipocalin-2	Lcn2	+1.9	+2.6	+6.3
complement component factor i	Cfi	+2.3	+1.3	+2.9
vascular cell adhesion molecule 1	Vcam1	+2.9	**−**1.1 (NS)	+4.5
guanylate binding protein 2	Gbp2	+2.1	+1.2 (NS)	+3.4
tumor-associated calcium signal transducer 2	Tacstd2	**−**2.4	**−**1.2 (NS)	+1.6
lectin, galactoside-binding, soluble, 3 binding protein	Lgals3bp	**−**3.4	**−**1.5	+1.8
phospholipase A2, group VII	Pla2g7	**−**1.1 (NS)	+1.4	**−**1.2 (NS)
immediate early response 3	Ier3	**−**1.2 (NS)	+2.0	+2.5
transporter 1, ATP-binding cassette, sub-family B	Tap1	**−**1.2 (NS)	**−**1.2 (NS)	+1.1 (NS)
receptor (calcitonin) activity modifying protein 2	Ramp2	**−**1.7	+1.1 (NS)	+1.5
phospholipid scramblase 1	Plscr1	+1.5	**−**1.2 (NS)	+2.1
**Decreased in microarray comparative analysis**				
deoxyribonuclease I	Dnase1	**−**2.4	+1.5	**−**8.1
carbonic anhydrase 14	Car14	**−**1.4	+1.3	**−**1.9
Afamin	Afm	+1.0 (NS)	+1.0 (NS)	**−**2.5
αKlotho	α-Kl	+1.9	+1.5	+1.0 (NS)
angiotensin I converting enzyme 2	Ace2	**−**16.7	(**−**1.3) NS	**−**15.6
solute carrier family 2, member 2	Slc2a2	**−**2.2	**−**1.1 (NS)	**−**4.5
thioesterase superfamily member 2	Them2	**−**1.1 (NS)	**−**1.2 (NS)	**−**1.9

Values are expressed as mean±SEM and as a relative fold change of the non injected Fgf23^−/−^ mice control value. Single (Fgf23^−/−^) and compound (Col4a3^−/−^Fgf23^−/−^) mice were injected (+rFGF23) or not twice a day with 50 ng/g of recombinant rat FGF23 during 8 weeks. Comparisons were performed using Student T test. P<0.05 vs. Ctr. N≥3/group. NS, gene is present but is not significantly different.

Secondly, we have transferred FGF23^−/−^ mice on the Col4a3^−/−^ background, thus identifying genes that respond to CKD progression independently of FGF23. We found that lcn2, cfi, pla2g7, and ier3 were upregulated by kidney disease progression. In addition, we also administered rFGF23 to compound FGF23^−/−^Col4a3^−/−^ mice, to attempt separation of CKD effects from those mediated by FGF23, as well as possible interactions between FGF23 and CKD. The transfer of FGF23^−/−^ on the CKD background, singled out genes that respond to FGF23 only with decline in renal function (upregulated: tacstd2, lgals3bp, ramp2; downregulated: afm, them2). Renal failure and FGF23 interacted to further increase FGF23 actions on lcn2, cfi and ier3, while CKD, although without independent regulatory actions per se, potentiated the effects of FGF23 on gbp2,,plscr1, dnase1 and slc2a2. Interestingly, Klotho is upregulated by rFGF23 in FGF23^−/−^ mice and normalized in animals with impaired renal function.

Since the chronic admnistaration of FGF23 may lead ot systemic changes, we also evaulated the rapid, short-term response to rFGF23 admnistration. This was accomplished by examining the acute effects of rFGF23 administration in C57Bl6 mice after 1 and 12 h. We found that injection of rFGF23 resulted in a 10-fold increase in Cyp24a1 one hour after injection that persisted after 12 h. We also observed that rFGF23 induced a decrease in Cyp27b1 and Npt2c, but had no effect on Npt2a (**Table 7**). Lipocalin2 was confirmed to be increased in the kidney following rFGF23 administration. We also found that GBP2 Tacstd2 and Plscr1 were increased in response to acute FGF23 elevation. Furthermore, Dnase1 and Car14 were decreased by FGF23, consistent with the microarray data. However, we could not confirm FGF23 regulation of ACE2 and Them2 in these short-term FGF23 administration studies.

**Table 7:** Expression fold change of renal genes modified 1 and 12 h after injection or rFGF23.

Gene Name	Symbol	1 h	12 h
**FGF23 regulated genes involved in mineral metabolism**			
**cytochrome P450, family 24, subfamily A, polypeptide 1**	**Cyp24a1**	**10.8**	**3.5**
**cytochrome P450, family 27, subfamily B, polypeptide 1**	**Cyp27b1**	**−3**	NS
**solute carrier family 34 (sodium phosphate), member 3**	**Npt2c**	**−1.3**	**−1.5**
**solute carrier family 34 (sodium phosphate), member 1**	**Npt2a**	**NS**	**NS**
**Increased in microarray comparative analysis**			
**Lipocalin-2**	**Lcn2**	**3.3**	NS
**complement component factor i**	**Cfi**	**2.2**	**3.1**
vascular cell adhesion molecule 1	Vcam1	NS	NS
**guanylate binding protein 2**	**Gbp2**	**2.2**	NS
**tumor-associated calcium signal transducer 2**	**Tacstd2**	**1.5**	**2.3**
**lectin, galactoside-binding, soluble, 3 binding protein**	**Lgals3bp**	NS	**2.3**
**phospholipase A2, group VII**	Pla2g7	1.4	**−**1.5
immediate early response 3	Ier3	NS	NS
transporter 1, ATP-binding cassette, sub-family B	Tap1	NS	NS
receptor (calcitonin) activity modifying protein 2	Ramp2	NS	NS
**phospholipid scramblase 1**	**Plscr1**	NS	**2**
**Decreased in microarray comparative analysis**			
**deoxyribonuclease I**	**Dnase1**	NS	**−2.0**
**carbonic anhydrase 14**	**Car14**	NS	**−2.2**
Afamin	Afm	NS	NS
**αKlotho**	**α-Kl**	**−1.5**	**1.7**
angiotensin I converting enzyme 2	Ace2	NS	NS
solute carrier family 2, member 2	Slc2a2	1.3	1.5
thioesterase superfamily member 2	Them2	NS	NS

Values are expressed as mean±SEM and as a relative fold change of the non injected control WT mice (Ctr)value. Comparisons were performed using Student T test. P<0.05 vs. Ctr. N≥ 4/group. NS, gene is present but is not significantly different.

To investigate if FGF23 directly regulates these genes, we examined the effects of rFGF23 on distal 209 renal tubular cells *in vitro*. By real-time PCR, we found that 209 cells express α-klotho and FGFR4, and lesser amounts of FGFR1 or FGFR3 transcripts (data not shown). We found that 8 of 10 genes tested were directly modified by FGF23 *in vitro*, including FGF23 stimulation of increments in Cf1 and Ramp1 and decrements in α-Klotho, Car14, Slc2a2, ACE2, DNAse 1, and Afm in distal tubule cells after treatment with FGF23 (**Table 8**).

**Table 8:** Expression fold change of selected genes confirmed by RT-PCR in a distal cell culture model, after 12 h of rFGF23 treatment.

Gene Symbol		Distal (209) Cells
Vcam1		NS
**Cfi**		**2.0**
Pla2g7		NS
**Ramp2**		**1.3**
**αKl**		**−1.8**
**Car14**		**−3.7**
**Slc2a2**		**−2.2**
**Ace2**		**−1.7**
**Dnase1**		**−1.4**
**Afm**		**−1.4**

Distal (209) tubular cell lines were cultured during 1 week and treated with 2 µg of rFGF23 per well. Values are expressed as mean±SEM and as a relative fold change of the untreated control. Comparisons were performed using Student T test. N≥ 4; P<0.05 vs: untreated control.

## Discussion

Comparative analysis of gene expression profiles of the Col4a3^−/−^ mice, a CKD model of elevated circulating levels of FGF23, and two other models of FGF23 excess and normal renal function [31], [40], [42], [43], along with confirmation of FGF23 regulation of these transcripts *in vivo* and *in vitro,* identified novel genes not previously recognized to be regulated by FGF23 as well as confirmed the regulation of genes known to be regulated by FGF23 in the kidney.

The effects of FGF23 on phosphate and vitamin D metabolism are mediated by the regulation of Npt2a, Cyp27b1 and Cyp24a1 functions in the proximal tubule [9]. With the exception of Npt2a, we have evidence in Col4a^−/−^ mice of alterations in Cyp24a1, Nap2a, Npt2c by FGF23, consistent with their known involvement in mediating FGF23 effects on kidney phosphate, calcium and vitamin D metabolism. The failure to observe changes in Npt2a gene transcripts likely points to the important role of post-translation regulation of brush border membrane insertion of this transporter in the regulation of phosphate transport [44]. Also, consistent with post-transcriptional regulation of Cyp27b1, FGF23 had only transient effects on Cyp27b1 gene transcription [45].

While serum soluble α-Klotho concentrations are inversely correlated with serum FGF23 [46] and reductions in α-Klotho mRNA levels in the kidney have been observed with CKD and other states of FGF23 excess FGF23 [47], direct regulation of α-Klotho by FGF23 has not been previously demonstrated. Rather, reductions in α-Klotho in CKD has been attributed to a primary decrement α-Klotho caused by loss of renal tubular cells in the diseased kidney, leading to secondary increments in FGF23 [48]. Both α-Klotho message and protein were decreased in kidneys of Col4a3^−/−^ mice, and the administration of rFGF23 results in decrements in α-Klotho expression in the kidney of wild-type mice and in cultured distal tubular cells. Interestingly however, chronic administration of rFGF23 to both Fgf23^−/−^ and Col4a3^−/−^Fgf23^−/−^ mice, failed to suppress α-Klotho message levels, which may be due to offsetting effects of 1,25(OH)_2_D, which is known to stimulate α-Klotho gene transcription [49], in this model. Regardless, FGF23 suppression of α-Klotho might have several physiological effects, including providing a mechanism to desensitize FGF23 signaling responses through FGFR [50] as well as regulate circulating forms of α-Klotho produced by the distal tubule that potential act as a hormone and/or paracrine co-factor for several growth factor receptors [28], [48] [48], [51].

We identified other gene products that could potentially account for the associations between elevated circulating FGF23 concentrations renal failure progression and cardiovascular mortality that have been found in clinical association studies. At present it is not certain if these untoward effects associated with elevations in FGF23 are due to direct effects of FGF23 on FGFR/α-Klotho complexes in the kidney, off “target effects” of high levels of FGF23 to directly activate FGFRs in the absence of α-Kotho in the heart [22], or represent epiphenomena caused by effects of CKD to increase FGF23 levels. In support of on-target actions, we identified FGF23-regulated renal genes with mechanistic linkages to cardiovascular diseases. In this regard, we found that ACE2 is reduced by excess FGF23 in all three models and found that FGF23 suppresses ACE2 expression in the distal tubule cultures. ACE2 is a negative regulator of the rennin-angiotensin system (RAS) that has vasodilator and natriuretic effects, leading to reduced blood pressure [52]. A direct effect of FGF23 to suppress ACE2 provides an alternative explanation for the recently proposed associations between vitamin D deficiency, activation of the renin-angiotensin system and regulation of α-Klotho expression [53]. FGF23 direct suppression of ACE2 expression could lead to activation of RAS, establishing a linkage between increased FGF23 and increased mortality.

We also identified renal gene products that may mediate a direct effect of FGF23 to accelerate the progression of chronic kidney disease. In addtion to α-Klotho, which has been shown to modulate renal damage [54], we also observed FGF23 regulation of several other genes associated with renal injury, including, Cfi, which has been shown to contribute to inflammatory and acute renal injury [55], [56], and suggesting an effect of FGF23-mediated complement activation; DNase1, which is associated with systemic lupus erythematosus (SLE) [57], [58], implicating a role of FGF23 in stimulating inflamatory responses in the kidney; carbonic anhydrase 14 (car14), whose inactivation in transgenic mice leads to progressive renal injury [59], [60]. Additionally, both slc2a2 and afamin were found to be downregulated in the distal tubular cell line by Fgf23. Slc2a2 (also known as the glucose transporter gene GLUT2) is a disease causing gene for Fanconi-Bickel syndrome, which has systemic as well as characteristic tubular nephropathy abnormalities [61]. Decrements in afamin (Afm), a vitamin E-binding protein, related to vitamin D binding protein [62], are observed in early acute renal allograft rejection [63].

Regarding additional genes involved in renal injury, we found that lcn2 (or NGAL) was markedly increased in the Col4a3^−/−^ and the FGF23Tg data set as well as in Fgf23^−/−^ and Col4a3^−/−^ Fgf23^−/−^ mice receiving rFGF23. Lcn2 mRNA is normally expressed in the kidney [64] where it promotes epithelial differentiation of the mesenchymal progenitors, leading to the generation of glomeruli, proximal tubules, Henle’s loop and distal tubules [65], [66]. Lcn2 expression is markedly induced in injured epithelial cells through NF-κB dependent pathways [67], and plays a central role in controlling cell survival and proliferation [68]–[69]. Lcn2 has been shown to contribute to CKD progression both in mice and humans [70]. The magnitude of the increase in Lcn2 as a response to FGF23 was greater in animals with CKD, suggesting that other factors other than FGF23 are also stimulating transcription of this gene. Additionally, 8 other genes which are significantly expressed during the repair stage in AKI were also increased in the kidneys of Col4a3^−/−^ mice, including C3, Vcam1, Serpin10 m C3, Lyz, Col3a1, Col1a1,and abp1 [71]. Finally, Them2, which belongs to a family of enzymes that play an important role in lipid metabolism and might contribute to tubular toxicity [72] was also increased in models of FGF23 excess.

Finally, pathway analysis identified TGF- β and TNF- α signaling pathways as being involved in FGF23 responses in the kidney. The TGF-β pathway are increased in most forms of CKD in humans and experimental animals and controls including fibrogenesis, apoptosis, epithelial-to-mesenchymal transition, and inflammation leading to glomerulosclerosis and tubulointerstitial fibrosis [73]. Three additional genes observed to be altered in a Tgf-β1 Tg mouse model of CKD, were also in the top modified genes in the Col4a3^−/−^ kidneys, including Timp1, Lcn2 and Cxcl1 [73]. TNF alpha is a central proinflammatory agonist mediator that is generated in a wide variety of innate and adaptive immune responses inflammatory mechanisms regulated by TNF might contribute to renal disease progression and cardiovascular events [74], [75], [76], and even in non-calcified aortas in patients with CKD display increased TNF immunoreactivity [77]. The central role of these pathways, together with IL1-beta, another pro-inflammatory cytokine, demonstrate that the inflammatory state that correlates with kidney disease may be modified by FGF23. We also found FGF23 associated increases in VCAM1, which is expressed in proximal tubule cells in response to inflammatory renal diseases [41], and interferon-induced guanylate-binding protein 2 (GBP2), which regulates cell growth and matrix metalloproteinase expression [78].

Our analysis has several limitations. Data sets from Hyp and FGF23Tg mice have fewer number of genes analyzed than the Col4a3^−/−^ (∼13,000 vs. ∼45,000, respectively), with the resulting possibility that some genes may have been missed. The presence of CKD could mask FGF23-responsive genes since both FGF receptors and klotho expression and function are altered. However, this appears to be an minor issue, since we confirmed that the known FGF23 regulated genes were still altered in this model. In addition, the microarray analysis was performed in whole tissues, which gives a composite read out of all cell types. We also did not define the specific tubular segments or the role of Klotho/FGFR complexes in the FGF23-mediated changes in gene expression in the kidney. Further studies will be needed to determine the cell-type specific alterations in gene expression. Age difference between the animals of all three databases may also confound the interpretation. The functional significance of FGF23 regulation of these genes remains to be established.

Regardless, we have discovered novel and potentially important FGF23 regulated genes involved in inflammation and progressive renal fibrosis as well as alterations in factors with systemic effects, such as ACE2, which might impact on cardiovascular function. Further studies are needed to test the role of these factors in linking FGF23 to mortality and progressive renal dysfuction.

## Materials and Methods

### Animals and Genotyping

All mice were maintained on a standard diet (7912, Harlan Teklad, Madison, WI, USA). Animal care and protocols were in accordance with the guidelines established by the University of Tennessee Institutional Animal Care and Use Committee as detailed in the “Guide for Care and Use of Laboratory Animals,” prepared by the Institute on Laboratory Animal Resources, National Research Council (Department of Health & Human Services Publication NIH 86–23, National Academy Press, 1996) and UTHSC IACUC specifically approved this study (protocol 1884). Animals were anesthetized before serum collection and sacrifice by ip injection of ketamin (120 mg/Kg) and xylazin (20 mg/Kg), followed by cervical dislocation. During the entire period of the study, activity, respiratory rate, muscle strength via grip strength, feeding and drinking, fur loss were the major signs and symptoms that have been monitored.three times a week by the investigator team and daily by the Comparative Medicine employees. If any signs of discomfort or infection were observed, the animal was euthanized by CO2 inhalation followed by cervical dislocation and excluded from the study.

Heterozygous Col4a3^+/−^ mice were initially obtained from Jackson Laboratories (Westgrove, PA, USA). To obtain the compound Col4a3^−/−^ Fgf23 mice ^−/−^ we first crossed heterozygous Col4a3^+/−^ females to Fgf23 mice ^+/−^ males to obtain Col4a3^+/−/^Fgf23^+/−^mice and then crossed Col4a3^+/−/^Fgf23^+/−^ males to Col4a3^+/−/^Fgf23^+/−^.

Tail or ear biopsies were collected to genotype the mice. REDExtract-N-Amp Tissue PCR Kit (Sigma-Aldrich, St. Louis, MO, USA) was used for DNA extraction and PCR amplification. Mice were genotyped for col4a3 mutation and PCR was repeated in all mice after sacrifice to exclude artifacts and ensure the correct genotype [30], [79].

### Administration of Rat Recombinant FGF23

Rat recombinant FGF23 (rFGF23) was administered intraperitoneally (ip) to WT, Fgf23^−/−^ and Col4a3^−/−/^Fgf23^−/−^ mice. To test the chronic effects of FGF23, FGF23^−/−^ and compound Col4a3^−/−^ FGF23^−/−^ mice were administered twice daily (every 12 hours) with 50 ng/g rFGF23 during eight weeks. Kidneys were collected 12 hours after the last rFGF23 administration. This procedure partially corrected the circulating FGF23 levels in serum samples collected 6 and 12 hours after the last injection (69±22 and 47±16 in FGF23^−/−^ mice and 65±14 and 41±12 in Col4a3^−/−^FGF23^−/−^). To test the acute effects of excess FGF23, C57Bl6 mice were given a single injection of 50ng/g rFGF23 and the kidneys were collected 1 and 12 hours after the injection. Experimental animals were compared to animals of the same genotype receiving 0.9% NaCl vehicle.

### Serum Biochemistry

Serum samples were collected by intracardiac exsanguination. Serum calcium was measured using a Calcium CPC Liquicolor Kit (Stanbio Laboratories, Boerne, TX, USA) and serum phosphorus was measured using the phosphomolybdylate-ascorbic acid method, as previously described [80]. Serum parathyroid hormone (PTH) levels were measured using the Mouse Intact PTH ELISA kit (Immutopics, Carlsbad, CA, USA). Serum 1,25(OH)_2_D and 25OHD levels were measured using the vitamin D EIA Kits (Immunodiagnostic Systems, Fountain Hills, AZ, USA). Serum FGF23 levels were measured using the FGF23 ELISA kit (Kainos Laboratories, Tokyo, Japan).

### RT-PCR and Microarray

RT-PCR and microarray analysis were performed on kidneys from 12 week-old mice. Total RNAs were isolated using TRI-reagent (Molecular Research Center, Cincinnati, OH, USA) according to previously published method [81]. First-strand cDNA was synthesized from the kidney RNAs using iScript cDNA Synthesis kit (Bio-Rad, Hercules, CA, USA). The 20µL reverse transcriptase reaction was based on 1µg total RNA. The iCycler iQ Real-Time PCR Detection System and iQ SYBR Green Supermix (Bio-Rad, Hercules, CA, USA) were used for real-time quantitative PCR analysis. The expression was normalized by glyceraldehyde-3-phosphate dehydrogenase (*Gapdh*) in the same sample and expressed as 100% of the control (WT). Sequences of primers used for real-time quantitative RT-PCR are listed in **Table 9**. The expression of 45,000 genes was tested on the kidney samples using the Illumina.SingleColor.MouseWG-6_V2_0_R1_11278593_A chip (Illumina, San Diego, CA, USA) at the DNA Discovery Core of University of Tennessee Health Science Center on 4 male mice per group. The resulting data were compared with previously published data reflecting the renal transcriptome in Hyp [40] and FGF23 transgenic mice [31].

**Table 9:** Sequences of primers used for RT-PCR.

Target Gene	Forward Primer	Reverse Primer
**Ace2**	CTTCTCTTCTCAGTGCCCAACCCA	CCCGTGCGCCAAGATCCCAT
**Adamts2**	CTGACGCCCAGGGCCGCTT	CGCCGTGAGCTGTTGATGCG
**Afm**	AGT GAC GAG TTC GCC TGC GT	CTG GCA CTG GCT TTG GTC GGT
**Aqp 11**	GTC CCC CGA AAT GGG TGC CG	GGC TCC CTC CTG CAT AGG CCA
**Car14**	TTG GAT CCT GGC TGC AGA TGG G	TGG CCA ATG GTC CTG ACC GTG
**Cfi**	AGA CTT GGC CCC GCA CTC CT	CAC ACA CTG GGG TGC CAG CC
**Cyp 51**	CCC TCA GAC GGT GGC AGG GT	GTC CAA GCG CTC TGC CCA GG
**Cyp24a1**	GTT CTG TCC ACG GTA GGC	CCA GTC TTC GCA GTT GTC C
**Cyp27b1**	ACA CTT CGC ACA GTT TAC G	TTA GCA ATC CGC AAG CAC
**Cyp2c44**	CCC AAG GGC ACC GCT GTG TT	AGC TCC ATG CGG GCC AAA CC
**Cyp2d12**	AGC CCA GAT CCC AAG GGC AGT	GGT GAC TGG GCA GGG TCC CA
**Dnase1**	TGC CTG GAC AGC GAC CCT GA	TGA GCC CCC GAG TCT GCA CT
**Gbp2**	ACA GTG CCT GTG AGA GAG GAC AGA	CTG TGC GGT AGA GGC CCA CGA
**Hbb-b1**	GCT TCT GAT TCT GTT GTG TTG ACT TGC	GAC AAC CAG CAG CCT GCC CA
**Hbb-b2**	AGG CCC TGG GCA GGT TGG TA	GCC ATG GGC CTT CAC CTT GGG
**Ier3**	GGC GCC AGC TAC CAA CCG AG	GAC CGG GGG CGC AGT AAT GG
**Lcn2**	TGG CAG GCA ATG CGG TCC AG	CCG TGG TGG CCA CTT GCA CA
**Lgals3bp**	AAG TGG TGG GCA GCA GCG TC	GCT CGA ACA GCT CCT GGG GC
**Mgp**	GCAGCGCCGAGGAGCCAAATA	AGGAAGGAGTGGGCCAGCCAG
**αKlotho**	AGC GAT AGT TAC AAC AAC	GCA TTC TCT GAT ATT ATA GTC
**Npt2a**	ATG CTG GCT TTC CTT TAC	CCA CAA TGT TCA TGC CTT CT
**Npt2c**	CGT GCG GAC TGT TAT CAA TG	TAC TGG GCA GTC AGG TTT CC
**Pla2g7**	TGC TGC CTC CCA TGG GTC CA	AGC CGG CAG CAG ACA TCA CC
**Plscr1**	GAG TCC CCT CTG CGA GGG AAA GC	CCC CGG TGG ACA GTT CAG TGG A
**Ramp2**	GAC AGC GTT GTG CCT CCC TCC	GCT GCA CCA GGG AGC AGT TCG
**Slc2a2**	CCA GCT TTG CAG TGG GCG GA	CCC AGG GCA CCC CTG AGT GT
**Slc34a2**	AAA TGC CCA GCC CAA CCC CG	GTC CGG CCA CTT TGC CTC CA
**STAT3s1**	CCCCGAAGCCGACCCAGGTA	TGCTGCAGGTCGTTGGTGTCA
**Tacstd2**	GCG ATG GCG ACC CGC TTT TG	GAC CCC GCC TGG GCC ATT TG
**Tap1**	GCC CTT GAG GCC TTA TCG GCG	ATG AGA CAA GGT TGC CGC TGC TG
**Them2**	TTT CTC CCG AGC ACG ACG CG	GGA GCA GCC GAG ACA AGC GT
**Timp1**	CAC GGG CCG CCT AAG GAA CG	TCC GTG GCA GGC AAG CAA AGT
**Vcam1**	TGT CAA CGT TGC CCC CAA GGA	GGC ATC CTG CAG CTG TGC CT

### Western Blotting and Immunohistochemistry

These techniques were performed as previously described [8], [82], [83]. Briefly, total proteins from kidneys were extracted in 1ml lysis buffer of T-PER Tissue protein extraction reagent (Pierce, IL, USA). supplemented with protease inhibitors (Roche Applied Science, IN, USA). Protein lysates (25 µg/sample) were reduced and extracted in LDS Sample buffer (Invitrogen, CA, USA) heated for 10 min at 70°C, migrated on NuPAGE Novex 10% Bis-Tris Gels (Invitrogen, CA, USA), and then analyzed by Western blotting using the ECL Advance WB Detection Kit (GE Healthcare, UK). The immunoreactive bands were visualized using enhanced chemiluminescence detection reagents (GE Healthcare, UK) on a Fluor-S Multi Imager (BioRad, CA, USA). Band intensities were determined by densitometry using ImageJ (NIH, USA).

For immunohistochemistry, left kidneys were dehydrated in absolute ethanol and embedded in paraffin. 5µm thick sections were cut on a rotary microtome. Sections were dried overnight on pre-charged pre-cleaned slides (VWR Scientific, PA, USA), deparaffinized and rehydrated. Nonspecific sites were blocked with 1X animal free blocker (Vector Laboratories Inc., CA, USA) and then sections were incubated with specific primary antibodies for 1 hour. An Immunohistological Vectastain ABC kit (Vector Laboratories Inc., CA, USA) was subsequently used for detection of the target protein and slides counterstained with DAPI, dehydrated and mounted with entellan. The following primary antibodies have been used: goat-raised anti-human Klotho (sc-22218), goat-raised anti-human Cyp27b1 (sc-49642), goat-raised anti-human Cyp24a1 (sc-32165), goat-raised anti-mouse lipocalin2 (sc-18698), goat-raised anti-mouse DNase1 (sc-19269) from Santa Cruz Biotechnology (Santa Cruz, CA).

### Cell Culture

Immortalized renal tubular cells were kindly donated by Peter Friedman [84]. Cells were plated in standard 25 cm^2^ flasks and allowed to grow until confluence. Cells were removed with 0.25% trypsin solution containing 0.02% EDTA (Sigma- Aldrich, St. Louis, MO, USA) and plated on 6-well plates for 7 days, then treated with 2 µg/well of rFGF23 or vehicle for 24 hours.

### Statistics

Differences among the two groups were tested by Student T test using the Statistica software (Statsoft, Tulsa, OK, USA). The differences were considered statistically significant at p<0.05.

Microarray analysis and filtering was performed as previously described [8]. Briefly, microarray data were analyzed using GeneSpring GX7.3 software (Agilent Technologies, Santa Clara, CA, USA). The Robust Multichip Averaging probe summarization algorithm was used to perform background correction, normalization, and probe summarization. Data were normalized per chip and per gene to the median. Genes were filtered to include only those that were expressed in at least one of the eight samples. The statistical analysis was performed using a one-way ANOVA followed by Benjamini-Hochberg multiple test correction assuming variances were equals to minimize the false positive discovery. P value was set at 0.05. Cluster analysis using a gene tree classification, Pearson correlation and average linkage was then performed to identify groups of genes for which the patterns of expression were similar. Pathway analysis was performed using the Ingenuity program (Ingenuity Systems, Redwood City, CA, USA) to match the identified genes of interest to already known broader networks of genes contained in the literature database.
